# Store-operated Ca^2+^ channels in airway epithelial cell function and implications for asthma

**DOI:** 10.1098/rstb.2015.0424

**Published:** 2016-08-05

**Authors:** Krishna Samanta, Anant B. Parekh

**Affiliations:** Department of Physiology, Anatomy and Genetics, Sherrington Building, Parks Road, Oxford OX1 3PT, UK

**Keywords:** Ca^2+^ signalling, store-operated Ca^2+^ channel, airway epithelia

## Abstract

The epithelial cells of the lung are at the interface of a host and its environment and are therefore directly exposed to the inhaled air-borne particles. Rather than serving as a simple physical barrier, airway epithelia detect allergens and other irritants and then help organize the subsequent immune response through release of a plethora of secreted signals. Many of these signals are generated in response to opening of store-operated Ca^2+^ channels in the plasma membrane. In this review, we describe the properties of airway store-operated channels and their role in regulating airway epithelial cell function.

This article is part of the themed issue ‘Evolution brings Ca^2+^ and ATP together to control life and death’.

## 1. Introduction

Atopic asthma is the most common chronic disease in children in the Western world, and the number of cases continues to grow. In the UK, the proportion of children with asthma and allergies, in general, is higher than most other European countries, imposing a serious clinical and economic burden on the National Health Service. There is no cure for the disease, and therapies are directed at controlling the severity of the symptoms. There has been little change in treatment for several years with corticosteroids being prescribed for prolonged management and long-lasting β2 agonists for more immediate relief through bronchodilation. However, lung function in 30–40% of patients shows no signs of clinical improvement with these treatments [1], and chronic exposure to corticosteroids has side effects, including type II diabetes, osteoporosis, dyspeptic disorders and cataracts [2].

A characteristic of chronic asthma is substantial remodelling of the airway wall [3]. Changes include an increase both in smooth muscle mass and sensitivity to contractile agents, subepithelial thickening owing to increased deposition of collagen and other components of the extracellular matrix below the epithelial basement membrane, the appearance of gaps between epithelial cells and hyperplasia of mucus-producing goblet cells [4].

The epithelial cells of the lung are at the interface of a host and its environment and are therefore directly exposed to inhaled air-borne particles. Although long considered to have a passive role in the airway remodelling process, growing evidence suggests that airway epithelial cells may make a more active contribution by reacting directly to allergens and triggering and then helping orchestrate the subsequent immune response [5].

Lung epithelial cells release a plethora of signals that recruit and activate immune cells that shape the subsequent inflammatory response. Epithelia-derived stimulants include ATP, uric acid, lysophosphatidic acid, granulocyte macrophage-colony stimulating factor, chemokine (C–C motif) ligand 2/20 (CCL2/CCL20) chemokine ligands, prostaglandin E_2_, thymic stromal lymphopoietin, regulated on activation, normal t expressed and secreted (RANTES) and various interleukins (ILs), including IL-1, -8 and -33 [6,7]. These signals target the components of both the innate and adaptive immune system, with important roles for antigen-presenting lung dendritic cells, mast cells and Th2 lymphocytes. In addition, various growth factors such as epidermal growth factor (EGF), amphiregulin and heparin-binding epidermal growth factor-like growth factor are secreted from airway epithelia and directly contribute to the remodelling process [7,8].

An important question concerns the mechanisms of activation of airway epithelial cells. Which intracellular second messenger pathways lead to the synthesis and secretion of the epithelia-derived signalling molecules? Identification of the underlying pathways may open up new potential therapies for manipulating epithelial cell activity and thereby impact on the remodelling process itself. As with many other cell types, it turns out that cytoplasmic Ca^2+^ is a critical intracellular signal in airway epithelia and this trigger Ca^2+^ is mainly derived from store-operated Ca^2+^ channels in the plasma membrane.

## 2. Ca^2+^-dependent responses in airway epithelia

**Figure 1.**
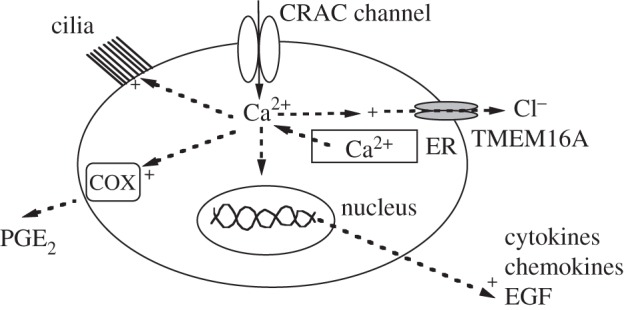
Cartoon summarizes various Ca^2+^-dependent responses that are activated by a rise in cytoplasmic Ca^2+^ in airway epithelia. Shown are an increase in ciliary beat frequency, cyclooxygenase activity (COX) and PGE_2_ secretion, activation of TMEM16A Cl^−^ channels, as well as increased gene expression of cytokines, chemokines and EGF. The Ca^2+^ rise is accomplished through both InsP_3_-driven Ca^2+^ release from the endoplasmic reticulum (ER) and Ca^2+^ influx through CRAC channels.

A rise in cytoplasmic Ca^2+^ can activate a range of temporally distinct responses in airway epithelial cells (figure 1).

Early work in type II airway epithelia showed that high concentrations of the Ca^2+^ ionophore A23187 led to an increase in secretion of pulmonary surfactant [9]. The secretagogue ATP, acting on P2Y receptors, also stimulated surfactant secretion and released Ca^2+^ from intracellular stores over a similar concentration range, suggestive of a causal relationship between cytoplasmic Ca^2+^ and surfactant secretion.

Airway mucus production is dependent on another form of secretion, namely that of Cl^−^. Recent work has found that the Ca^2+^-activated chloride channel TMEM16A is expressed in airway epithelial cells and is upregulated in asthmatics [10]. Studies with channel blockers revealed that TMEM16A contributed to both mucus secretion from epithelial cells and contraction of airway smooth muscle [10], the latter through depolarization of the membrane potential and subsequent opening of voltage-gated Ca^2+^ channels. Although TMEM16A channels are gated by cytoplasmic Ca^2+^, the EC_50_ for Ca^2+^ activation is strongly voltage-dependent, falling approximately 10-fold when the membrane potential changes from −100 to +100 mV [11]. At physiological potentials of around −80 to −40 mV, the EC_50_ for Ca^2+^ is around 2–4 µM. Because this is larger than the typical bulk cytoplasmic Ca^2+^ rise seen after stimulation of airway epithelia, trigger Ca^2+^ is probably from a local source such as Ca^2+^ entry through closely apposed store-operated Ca^2+^ channels in the plasma membrane (see below).

In cystic fibrosis, bacterial pathogens contribute to mucus hypersecretion through mobilization of intracellular Ca^2+^. Flagellin, a component of bacterial flagella, can bind to the glycolipid receptor asialoGM1, leading to interaction with Toll-like receptor 2 [12]. This results in secretion of ATP which then, in an autocrine loop, feeds back to activate phospholipase C-coupled P2Y receptors, resulting in Ca^2+^ release and activation of downstream Ca^2+^-dependent responses such as Ca^2+^-dependent Cl^−^ secretion.

Another important role for cytoplasmic Ca^2+^ in airway epithelial cells is to regulate ciliary beat frequency, an important step in clearance of mucus. ATP is an effective stimulus of ciliary beating while inducing numerous cytoplasmic Ca^2+^ oscillations [13]. Simultaneous measurement of ciliary beat frequency and either bulk cytoplasmic Ca^2+^ [14] or Ca^2+^ near the base of the cilia [15] showed that beat frequency was regulated by the frequency of Ca^2+^ oscillations induced by the stimulants acetylcholine or ATP, with high frequencies of Ca^2+^ signal inducing a steady elevated beat frequency [15]. Interestingly, these motile cilia also function as chemosensors in that they can detect and respond to inhaled bitter compounds such as denatonium and nicotine [16]. These compounds bind to bitter taste receptors of the T2R family, which are exclusively located on cilia. The receptors couple to phospholipase Ca^2+^ and, when activated, increase cytoplasmic Ca^2+^. This rise in Ca^2+^ was shown to increase ciliary beat frequency, providing a neat feedback loop whereby the cilia, having detected harmful particles, mechanically move the compounds out of the lung [16].

Ca^2+^ can also spread through airway epithelial cells as an intercellular Ca^2+^ wave following mechanical stimulation of a single cell. The Ca^2+^ wave can be propagated by inositol1,4,5-triphosphate (InsP_3_) [17], diffusing through gap junctions of connexin 32 proteins [18]. The spatial profile of the Ca^2+^ wave is significant, spreading to 40 cells or more following mechanical stimulation of just one cell [19]. An auxiliary mechanism could involve a paracrine signal, released in a Ca^2+^-dependent manner and which acts through G protein-coupled receptors that link to phospholipase C and thus produce InsP_3_. Possibilities include ATP or leukotriene C_4_, which acts on cysteinyl leukotriene type I receptors to drive intercellular Ca^2+^ waves in mast cells [20].

In addition to the relatively rapid onset responses described above, cytoplasmic Ca^2+^ can exert long-term effects on epithelial cell function through regulation of gene expression. Damage to airway epithelia such as mechanical or viral insults leads to remodelling and subsequent thickening of the airways and a major role for EGF in regulating the process has been established [21]. Insults to the airways also induce cytokine and chemokine transcription and secretion, important steps in the development of the ensuing immune response.

Ca^2+^ signals often regulate gene expression through activation of Ca^2+^-dependent transcription factors such as CREB and members of the NFAT family. NFAT proteins are heavily phosphorylated at rest and confined to the cytoplasm [22]. A cytoplasmic Ca^2+^ rise can activate the protein phosphatase calcineurin, the target for immunosuppressants cyclosporin A and tacrolimus, which dephosphorylates NFAT. Dephosphorylation leads to exposure of a nuclear localization sequence, resulting in nuclear import as part of a transport complex with importin β [23]. By contrast, Ca^2+^ entry activates nuclear CREB through a series of phosphoryation reactions [24]. Ca^2+^ microdomains near open CaV1 (L type) channels activate βCa^2+^/calmodulin-dependent protein kinase II (CaMKII) which is held close to the channel pore. Active βCaMKII then phosphorylates γCaMKII on threonine 287. Once phosphorylated, γCaMKII acts as a shuttle, transporting Ca^2+^–CaM into the nucleus. Nuclear Ca^2+^–CaM then activates Ca^2+^/calmodulin-dependent protein kinase kinase that in turn activates CaMKIV, resulting in CREB phosphorylation and transcription of CRE-regulated genes [24].

## 3. Sources of Ca^2+^ in airway epithelia

Resting free cytoplasmic Ca^2+^ concentration in most mammalian cells is typically 50–100 nM. However, high concentrations of free Ca^2+^ (hundreds of micromolar to millimolar) are stored in intracellular organelles such as the endoplasmic reticulum and Golgi apparatus (reviewed in [25,26]). These Ca^2+^ stores can be mobilized rapidly by second messengers such as InsP_3_ and cyclic ADP ribose, which target InsP_3_ receptor channels and ryanodine-sensitive Ca^2+^ release channels, respectively. Cytoplasmic Ca^2+^ itself is an important regulator of both these types of release channel, through both positive and negative feedback. The stores refill through Sarco/endoplasmic reticulum Ca^2+^ATPase (SERCA) and Golgi-located secretory pathway Ca^2+^ATPase (SPCA) pumps. Mitochondria, by virtue of the large negative potential across the inner mitochondrial membrane, are able to take up large amounts of cytoplasmic Ca^2+^ rapidly though the mitochondrial uniporter channel and then release the Ca^2+^ more slowly through Na^+^–Ca^2+^ exchange [27,28]. In addition, lysosomes and secretory vesicles can also function as sources of Ca^2+^ after stimulation [29].

Airway epithelia have a prominent endoplasmic reticulum from which Ca^2+^ can be rapidly released by agonists that couple to phospholipase C and thereby increase InsP_3_ levels. Treatment with a maximally effective concentration of the SERCA pump inhibitor thapsigargin, which gradually depletes stores of Ca^2+^, resulted in substantial Ca^2+^ release consistent with a large pool of Ca^2+^ contained within the organelle [30]. However, subsequent exposure to ATP still resulted in detectable Ca^2+^ release (*ca* 15% of control), despite the reduction in Ca^2+^ content of the endoplasmic reticulum. Although the endoplasmic reticular Ca^2+^ store clearly makes a major contribution to the pool of mobilizable Ca^2+^, the small thapsigargin-insensitive component to the ATP response led to the suggestion of an additional store, although its identity was unclear. However, in that study, ATP was applied when Ca^2+^ had barely returned to basal levels, raising the possibility that stores might not have been fully depleted by thapsigargin prior to ATP challenge. In addition to InsP_3_ receptors, many cell types co-express ryanodine-sensitive Ca^2+^ release channels. However, neither caffeine nor ryanodine, agonists of these channels, had any effect on cytoplasmic Ca^2+^ in rabbit tracheal epithelial cells [31].

Although Ca^2+^ release from the endoplasmic reticulum is important, the limited capacity of the store means that the Ca^2+^ mobilization phase is transient. To sustain Ca^2+^ signalling, Ca^2+^ influx into the cell is necessary. With an external free Ca^2+^ concentration in the plasma of *ca* 1.5 mM and a typical resting membrane potential of −70 mV, the electrochemical gradient for Ca^2+^ entry is large. Therefore, raising membrane permeability by increasing the open probability of Ca^2+^ channels, is a very effective way to increase Ca^2+^ flux into the cytoplasm. Airway epithelia, like most other non-excitable cells, do not express functional voltage-gated Ca^2+^ channels. Ca^2+^ influx to agonists are unaffected by CaV1.2 (L type) inhibitors such as verapamil and diltiazem [30]. Instead, transient receptor potential channels and store-operated Ca^2+^ entry provide the main routes for Ca^2+^ influx.

## 4. Store-operated Ca^2+^ entry

A major conduit for Ca^2+^ influx in non-excitable cells is through store-operated Ca^2+^ channels in the plasma membrane [32]. These channels are opened following the emptying of the endoplasmic reticular Ca^2+^ store [33]. Store depletion is accomplished physiologically by stimulation of cell-surface receptors that increase the activity of the enzyme phospholipase C, generating InsP_3_ following hydrolysis of the membrane phospholipid phosphatidylinositol-4,5-bisphosphate. Both G protein-coupled and tyrosine kinase-coupled receptors have been shown to activate store-operated channels [34]. Electrophysiological studies defined a highly Ca^2+^-selective, low conductance channel called the Ca^2+^ release-activated Ca^2+^ (CRAC) channel that was activated, regardless of how the Ca^2+^ store was emptied [33]. CRAC channels are widely expressed and are encoded by the Orai genes [35,36]. The channels are gated by direct physical interactions with stromal interaction molecules (STIM) 1 and 2, which function as endoplasmic reticulum Ca^2+^ sensors [37]. Upon a fall in endoplasmic reticulum Ca^2+^ content, STIM proteins multimerize and translocate to junctional endoplasmic reticulum that are located less than 20 nm from the plasma membrane. At these sites, they bind to and open the Orai channels, resulting in Ca^2+^ entry. The crystal structure of *Drosophila* Orai has been reported at 3.35 Å resolution [38]. The channel comprises a hexamer of Orai subunits arranged around a central pore. Loss-of-function mutations in either STIM1 or Orai1 genes are tightly linked to various forms of severe combined immunodeficiency [39,40]. Gain-of-function mutations in STIM1 and Orai1 have been reported and are associated with Stormorken syndrome, a rare disorder that results in thrombocytopenia, tubular aggregate myopathy, miosis, asplenia, ichthyosis and dyslexia [41].

The function of CRAC channels has been studied in detail in immune cells such as mast cells and T lymphocytes [42,43]. In mast cells, CRAC channels stimulate secretion [44], activate enzymes that lead to de novo synthesis and secretion of pro-inflammatory leukotrienes [45,46], increase expression of immediate early genes such as c-Fos [47,48], and various chemokines and cytokines that help orchestrate the subsequent inflammatory response [44]. In many of the above cases, spatially restricted Ca^2+^ signals (Ca^2+^ microdomains) near open CRAC channels and not a bulk rise in cytoplasmic Ca^2+^ are essential for activating these processes [46–48]. Ca^2+^ microdomains near CRAC channels [49,50] or CaV1.2 (L type) Ca^2+^ channels [51] have a privileged line of communication with NFAT, through association with members of the A-kinase anchoring protein (AKAP) family. AKAPs bind calcineurin and a fraction of the total NFAT pool, holding these proteins at the plasma membrane. CaV1.2 is associated with AKAP at rest [51], whereas for CRAC channels, the association is induced after store depletion [50]. In both cases, Ca^2+^ microdomains now have a direct line to calcineurin and NFAT, resulting in transcription factor activation in the absence of a bulk Ca^2+^ rise. Once within the nucleus, NFAT helps regulate gene transcription, often through interaction with the AP-1 complex [52], a heterodimer of the immediate early genes c-Fos and jun. Work in mast cells has shown that c-Fos expression is also increased by Ca^2+^ microdomains near CRAC channels [47]. This pathway is independent of AKAP/calcineurin [53] but requires the non-receptor tyrosine kinase Syk and the transcription factor STAT5 [47]. Hence, Ca^2+^ microdomains that have built up near CRAC channels signal through different pathways to regulate the activities of different transcription factors, which in turn can operate independently or synergistically.

## 5. Store-operated Ca^2+^ entry in airway epithelia

### (a) Functional store-operated Ca^2+^ influx

Measurements of cytoplasmic Ca^2+^ using fluorescent dyes have revealed that airway epithelia possess functional store-operated Ca^2+^ channels. Challenge of human bronchial epithelia 16-HBE cells with thapsigargin in Ca^2+^-free solution resulted in Ca^2+^ store depletion as the Ca^2+^ that flowed out of the store through the Ca^2+^ leak pathway was no longer taken back up by SERCA pumps. Readmission of external Ca^2+^ a few minutes later resulted in a robust cytoplasmic Ca^2+^ rise as Ca^2+^ entered the cell through the open CRAC channels [54]. In human bronchial epithelial cells from Lonza, activation of protease-activated receptor 2 (PAR2) with trypsin or P2Y2 and P2Y4 receptors with ATP or UTP resulted in prominent store-operated Ca^2+^ entry, following InsP_3_-dependent Ca^2+^ release from the stores [55]. Stimulation of either bradykinin or PAR1, 3, 4 receptors also resulted in Ca^2+^ influx but to a lesser extent. Therefore, as in other cell types, activation of Gq-coupled receptors results in store-operated Ca^2+^ influx in response to physiologically relevant stimuli.

### (b) STIM and Orai proteins in airway epithelia

In 16-HBE cells, STIM1 and STIM2 proteins are both expressed as are mRNA transcripts for Orai1 and Orai3 and to a lesser extent Orai2 [54]. Orai1 protein is also detectable. STIM1 and Orai1 proteins are likewise expressed in normal human bronchial epithelial cells [55]. Hence, airway epithelia express the two key components of the CRAC channel.

Knockdown of STIM1 using an siRNA-based approach resulted in a reduction in protein expression of 60% and this led to a substantial decrease in store-operated Ca^2+^ influx in response to thapsigargin [54]. Knockdown of STIM1 also reduced Ca^2+^ entry in response to PAR2 receptor activation [55]. Similarly, knockdown of Orai1 also severely abrogated store-operated Ca^2+^ entry [54]. STIM1 and Orai1 are therefore expressed and functional in airway epithelia.

### (c) Pharmacology of airway epithelia store-operated channels

The rise in cytoplasmic Ca^2+^ that occurred following readmission of external Ca^2+^ to cells challenged with thapsigargin in Ca^2+^-free solution was blocked by structurally distinct CRAC channel blockers including Synta66, BTP2, 2-APB as well as the rare earth element La^3+^ [54]. Inhibition of Orai1 by La^3+^ is reduced by mutating negatively charged aspartates (D110 and D112) in the extracellular loop between transmembrane domains I and II [56], which are close to the critical glutamate residue (E106) that confers Ca^2+^ selectivity [57]. If the airway epithelial CRAC channel pore is identical to that found in immune cells, then a prediction would be that CRAC channels in both cell types should show superimposable dose–inhibition curves to La^3+^. This was indeed the case [54]; the IC_50_ and Hill coefficient for La^3+^ block of CRAC channels in airway epithelia (0.8 and 1.0 µM, respectively) were very similar to that seen in the RBL-1 mast cell line (1.1 and 1.0 μM).

### (d) Electrophysiological properties of the store-operated channels

CRAC channels have several biophysical characteristics that combine to form a useful molecular fingerprint. These include its tiny unitary conductance (in the range of a few femtoSiemens in physiological Ca^2+^), steep inward rectification and high selectivity for Ca^2+^ [58]. The permeability ratio for Ca^2+^ to Na^+^ is greater than 1000 [59]. In the absence of external divalent cations however, the channels lose their exquisite selectivity for Ca^2+^ and now support large Na^+^ fluxes [59–61]. Another common feature of mammalian CRAC channels is the property of fast Ca^2+^-dependent inactivation, which develops bi-expontially with time constants of approximately 10 and approximately 100 ms [59,62,63]. Fast inactivation arises through a negative feedback pathway in which permeating Ca^2+^ ions reduce further Ca^2+^ entry. Studies investigating the impact of Ca^2+^ chelators with different on-rates on the kinetics and extent of fast inactivation suggest the incoming Ca^2+^ ions act within a few nanometres of the channel mouth [62,63].

Whole cell patch clamp experiments show that the airway epithelial store-operated current has numerous features characteristic of the CRAC channel. Store depletion following dialysis with InsP_3_ led to an inwardly rectifying Ca^2+^ current with a very positive reversal potential (greater than +70 mV), indicative of high selectivity for Ca^2+^ [54]. The store-operated current is blocked by the CRAC channel blocker Synta66 [54]. Moreover, the rate and extent of fast Ca^2+^-dependent inactivation are identical for CRAC channels in airway epithelia and RBL-1 mast cells, over a wide voltage range [54].

In the absence of external Ca^2+^ and Mg^2+^, the inward current became larger and the current–voltage relationship now reversed at +50 mV [55], consistent with monovalent permeation in the absence of divalent cations. Interestingly, the process of depotentiation of CRAC channels, whereby channel activity falls in divalent-free solution, appears less pronounced in airway epithelia than T cells, for example. The current in divalent-free solution falls around 40% after 20 s in divalent-free solution in T cells [60], whereas the reduction is around 10% in epithelia [55], although this was not quantified. Further work is therefore needed to see whether this is indeed a real difference. Interestingly, Orai3 shows little depotentiation in divalent-free solution [64], and Orai3 message is prominent in airway epithelial cells. It will be interesting to address the role, if any, of Orai3 in epithelial store-operated Ca^2+^ influx.

Nevertheless, several important features of the airway epithelial CRAC channel pore, channel pharmacology and regulation by Ca^2+^ closely match the channel robustly expressed in immune cells.

### (e) Airway epithelial store-operated Ca^2+^ channels activate gene expression

A major role for CRAC channels in airway epithelia is to regulate gene transcription. Expression of the immediate early gene c-Fos was increased approximately sixfold following stimulation with thapsigargin and this was suppressed by the CRAC channel blockers Synta66 and BTP2 [54]. c-Fos, as part of the AP-1 complex, is an important transcriptional regulator, often in tandem with NFAT. NFATc1 and NFATc3 mRNA (NFAT2 and NFAT4) were strongly expressed in alveolar epithelial type II cells, where the proteins were found to help regulate expression of surfactant protein D [65]. 16-HBE cells expressed both NFAT1 and NFAT4 mRNA, with the latter at approximatley twice the level of NFAT1 [54]. NFAT-driven green fluorescent protein reporter gene expression was increased several fold by thapsigargin and this was completely suppressed by CRAC channel blockers [54]. Consistent with this, thapsigargin increased the activity of recombinant luciferase under an NFAT promoter, and this was inhibited by BTP2 [55].

Activation of PAR2 receptors in airway epithelial cells induced expression of the cytokines interleukin (IL)-6 and IL-8 [55]. This increase was partially reduced by cyclosporin A, implicating a role for NFAT protein in regulation of these genes. Stimulation with the combination of thapsigargin and phorbol ester increased production of TNF-α, IL-6, IL-8 and PGE_2_, effects that were all reduced by cyclosporin A. Whereas cyclosporin A abolished IL-6 and TNF-α production, it only partially reduced IL-8 and PGE_2_ production. By contrast, IL-6 generation to PAR2 receptor activation was only moderately reduced by cyclosporine A, and PGE2 production was not compromised at all [55]. These differences suggest that, in contrast to thapsigargin stimulation, production of IL-6 is only partially NFAT-independent and PGE_2_ production is NFAT-independent following PAR2 receptor activation. PGE_2_ production following PAR2 receptor activation was partially sensitive to BTP2 suggesting that PGE2 production, although insensitive to NFAT, nonetheless required store-operated Ca^2+^. The underlying mechanism could involve Ca^2+^ entry through CRAC channels activating Ca^2+^-dependent phospholipase A_2_. This would liberate arachidonic acid, the substrate for the cyclooxygenase enzymes that lead to the production of prostaglandins. Such a mechanism has previously been described in mast cells, where local Ca^2+^ entry through CRAC channels activated the MEK/ERK pathway, which led phosphorylation and activation of Ca^2+^-dependent phospholipase A_2_ as well as stimulation of the 5-lipoxygenase enzyme [45,66]. Through this form of metabolic coupling orchestrated by ERK, the 5-lipoxygenase rapidly converted arachidonic acid released from phospholipids by Ca^2+^-dependent phospholipase A_2_ to leukotriene C_4_.

### (f) Ca^2+^ release-activated Ca^2+^ channels, epidermal growth factor and airway remodelling

Genetic linkage studies in asthmatic patients revealed a region on chromosome 7 containing the EGF receptor that was associated with airway hyper-reactivity [67], a step involved in the airway remodelling process. Furthermore, a study in Japanese asthmatics found that CA repeat polymorphisms in intron 1 of the EGF receptor were associated with the severity of the disease [68]. In biopsies taken from asthmatic subjects, the extent of immunostaining of the EGF receptor correlated with the pathology of the disease. A twofold increase was seen in mild forms of asthma, but this increased to almost threefold in more severe cases [69]. Collectively, these studies point to an important role for the expression of the EGF receptor in airway modelling that is characteristic of asthma. Functional studies strongly support this view. Ovalbumin-induced remodelling in rodent airways include airway smooth muscle hyperplasia, and epithelial and Goblet cell proliferation. Pre-treatment with AG1478, an EGF receptor inhibitor, prevented these changes from taking place [70]. Recently, heparin-binding epidermal growth factor was found to increase airway smooth muscle thickening in the ovalbumin-induced asthmatic mouse model. The growth factor also increased migration of airway smooth muscle in a Boyden chamber assay [71]. Collectively, these studies clearly document an important role for the EGF receptor in airway remodelling. In 16-HBE cells, transcription of EGF was increased following stimulation with thapsigargin, and this was suppressed by knockdown of either Orai1 or STIM1 [54]. Detectable basal EGF transcription was resolvable in the absence of stimulation, consistent with a tonic role for low levels of EGF in airway function. Although knockdown of STIM1 or Orai1 suppressed the increase in EGF transcription to thapsigargin, this was not reduced beyond the basal level [54]. This could indicate either the presence of an alternative Ca^2+^ entry pathway or that sufficient levels of STIM1/Orai1 remain after knockdown to maintain basal EGF production.

## 6. Conclusion

Airway epithelial cells lining the respiratory tract not only provide a first line of defence to air-borne pathogens and irritants, but also play a central role in initiating and orchestrating subsequent innate and adaptive immune responses. In addition to these physiological functions, airway epithelial cells contribute to the remodelling process characteristic of chronic asthma.

Epithelial cells release cytokines as well as a plethora of other bioactive compounds including EGF and related signalling molecules that are closely associated with the remodelling mechanism. Synthesis and release of many of these signals is triggered by Ca^2+^ entry through store-operated CRAC channels. These channels share several traits with their well-defined counterparts in mast cells and T lymphocytes, which help maintain the immune response in asthma. Therefore, targeting CRAC channels is a new and exciting approach to managing chronic airway diseases. By modulating activities of airway epithelial and mast/T cells at the same time, CRAC channel inhibitors would be particularly effective in damping down both immune and remodelling components. Although there are currently no CRAC channel blockers in use in the clinic, the area is extremely active with both small molecule inhibitors [72–74] and biologics [75] being pursued. Therefore, it is a realistic hope that in the near future CRAC channel blockers may be added to the clinical arsenal of drugs aimed at managing asthma.
